# Supplementary material for: Hot spot prediction in protein-protein interactions by an ensemble system
Source: BMC Syst Biol. 2018 Dec 31;12(Suppl 9):132. doi: 10.1186/s12918-018-0665-8 (PMC6311905; doi:10.1186/s12918-018-0665-8)
Supplement: Supplementary file 2 — All relevant MATLAB code. A simple Matlab implement of our predictor. (ZIP 19,742 kb) [file 12918_2018_665_MOESM2_ESM.zip › Additional file 2/Read ASA/ASA Property/BID/1EBPA.txt]

 NetSurfP - Protein Surface Accessibility and Secondary Structure Predictions

  

|  |  |  |
| --- | --- | --- |
|  |  | NetSurfP - Protein Surface Accessibility and Secondary Structure Predictions  Technical University of Denmark |

  


---

```
# For publication of results, please cite:
# A generic method for assignment of reliability scores applied to solvent accessibility predictions.
# Bent Petersen, Thomas Nordahl Petersen, Pernille Andersen, Morten Nielsen and Claus Lundegaard
# BMC Structural Biology 2009, 9:51 doi:10.1186/1472-6807-9-51
#
# Column 1: Class assignment - B for buried or E for Exposed - Threshold: 25% exposure, but not based on RSA
# Column 2: Amino acid
# Column 3: Sequence name
# Column 4: Amino acid number
# Column 5: Relative Surface Accessibility - RSA
# Column 6: Absolute Surface Accessibility
# Column 7: Z-fit score for RSA prediction
# Column 8: Probability for Alpha-Helix
# Column 9: Probability for Beta-strand
# Column 10: Probability for Coil
E K  1EBPA                  1    0.813 167.275   1.377   0.003   0.003   0.994
B F  1EBPA                  2    0.298  59.889  -0.672   0.021   0.279   0.699
E E  1EBPA                  3    0.507  88.625   0.975   0.022   0.552   0.426
E S  1EBPA                  4    0.525  61.483   0.034   0.021   0.451   0.528
E K  1EBPA                  5    0.508 104.434   1.088   0.069   0.386   0.545
E A  1EBPA                  6    0.431  47.496  -0.469   0.066   0.296   0.638
E A  1EBPA                  7    0.502  55.364  -0.240   0.069   0.386   0.545
E L  1EBPA                  8    0.369  67.472  -0.836   0.069   0.386   0.545
B L  1EBPA                  9    0.255  46.727  -0.048   0.064   0.216   0.721
B A  1EBPA                 10    0.216  23.781   0.057   0.018   0.088   0.893
E A  1EBPA                 11    0.347  38.217  -0.583   0.018   0.047   0.935
E R  1EBPA                 12    0.751 171.979  -0.368   0.018   0.019   0.964
E G  1EBPA                 13    0.612  48.149   0.533   0.018   0.019   0.964
B P  1EBPA                 14    0.139  19.752  -0.740   0.005   0.015   0.979
E E  1EBPA                 15    0.581 101.553   0.345   0.018   0.047   0.935
E E  1EBPA                 16    0.506  88.398   0.488   0.004   0.138   0.858
B L  1EBPA                 17    0.059  10.840   0.502   0.005   0.336   0.660
E L  1EBPA                 18    0.298  54.619   0.696   0.003   0.718   0.279
B C  1EBPA                 19    0.029   4.086  -0.699   0.001   0.900   0.099
B F  1EBPA                 20    0.207  41.545   1.387   0.001   0.959   0.040
B T  1EBPA                 21    0.119  16.505  -0.059   0.001   0.959   0.040
E E  1EBPA                 22    0.347  60.708   0.727   0.002   0.816   0.182
E R  1EBPA                 23    0.412  94.394  -0.327   0.004   0.197   0.799
E L  1EBPA                 24    0.384  70.384   0.628   0.004   0.197   0.799
E E  1EBPA                 25    0.396  69.216   0.212   0.003   0.718   0.279
E D  1EBPA                 26    0.278  39.988   0.925   0.001   0.900   0.099
B L  1EBPA                 27    0.031   5.713   0.377   0.001   0.900   0.099
B V  1EBPA                 28    0.251  38.609   1.337   0.001   0.959   0.040
B C  1EBPA                 29    0.017   2.345   1.339   0.001   0.959   0.040
B F  1EBPA                 30    0.220  44.114   1.183   0.001   0.959   0.040
B W  1EBPA                 31    0.049  11.857  -0.063   0.001   0.900   0.099
E E  1EBPA                 32    0.498  86.913   0.891   0.004   0.616   0.381
E E  1EBPA                 33    0.370  64.604   0.699   0.004   0.138   0.858
E A  1EBPA                 34    0.452  49.766  -0.768   0.005   0.015   0.979
E A  1EBPA                 35    0.658  72.567  -0.718   0.005   0.015   0.979
E S  1EBPA                 36    0.647  75.817  -1.825   0.005   0.045   0.951
E A  1EBPA                 37    0.435  47.904  -1.107   0.004   0.085   0.910
E G  1EBPA                 38    0.582  45.843  -2.422   0.005   0.045   0.951
E V  1EBPA                 39    0.396  60.881  -2.550   0.005   0.045   0.951
E G  1EBPA                 40    0.477  37.579  -0.327   0.004   0.138   0.858
E P  1EBPA                 41    0.313  44.401  -0.987   0.004   0.420   0.576
E G  1EBPA                 42    0.304  23.964  -0.275   0.004   0.616   0.381
B N  1EBPA                 43    0.259  37.874  -0.144   0.002   0.816   0.182
B Y  1EBPA                 44    0.053  11.262   0.732   0.001   0.900   0.099
B S  1EBPA                 45    0.171  20.018   0.807   0.001   0.959   0.040
B F  1EBPA                 46    0.033   6.583   0.356   0.001   0.959   0.040
B S  1EBPA                 47    0.090  10.536   0.597   0.001   0.959   0.040
B Y  1EBPA                 48    0.079  16.840   0.032   0.001   0.959   0.040
B Q  1EBPA                 49    0.225  40.256   0.030   0.001   0.900   0.099
B L  1EBPA                 50    0.256  46.819   0.189   0.003   0.718   0.279
E E  1EBPA                 51    0.584 101.972  -0.600   0.020   0.205   0.775
E D  1EBPA                 52    0.721 103.925  -1.315   0.018   0.019   0.964
E E  1EBPA                 53    0.644 112.507  -0.775   0.018   0.047   0.935
E P  1EBPA                 54    0.587  83.366   0.511   0.020   0.205   0.775
E W  1EBPA                 55    0.382  91.799  -0.040   0.021   0.451   0.528
E K  1EBPA                 56    0.581 119.553   0.640   0.021   0.451   0.528
E L  1EBPA                 57    0.546  99.973   0.880   0.022   0.359   0.619
B C  1EBPA                 58    0.076  10.642  -0.827   0.019   0.141   0.840
E R  1EBPA                 59    0.558 127.782  -0.077   0.004   0.197   0.799
E L  1EBPA                 60    0.423  77.433  -1.020   0.004   0.420   0.576
E H  1EBPA                 61    0.330  60.063   0.136   0.023   0.655   0.322
E Q  1EBPA                 62    0.459  81.995  -0.783   0.021   0.756   0.223
E A  1EBPA                 63    0.455  50.141  -0.450   0.021   0.756   0.223
E P  1EBPA                 64    0.501  71.106  -1.612   0.021   0.451   0.528
E T  1EBPA                 65    0.379  52.623  -0.704   0.004   0.138   0.858
E A  1EBPA                 66    0.580  63.971  -1.683   0.005   0.045   0.951
E R  1EBPA                 67    0.528 120.820  -1.498   0.004   0.085   0.910
E G  1EBPA                 68    0.531  41.813  -1.445   0.004   0.085   0.910
E A  1EBPA                 69    0.423  46.560  -1.465   0.005   0.336   0.660
E V  1EBPA                 70    0.293  45.080  -0.666   0.004   0.616   0.381
E R  1EBPA                 71    0.379  86.768  -0.261   0.002   0.816   0.182
B F  1EBPA                 72    0.153  30.807  -0.563   0.002   0.816   0.182
B W  1EBPA                 73    0.255  61.231  -0.671   0.002   0.816   0.182
B C  1EBPA                 74    0.027   3.819  -0.777   0.001   0.900   0.099
E S  1EBPA                 75    0.300  35.137   0.370   0.001   0.900   0.099
B L  1EBPA                 76    0.046   8.368  -0.078   0.002   0.816   0.182
E P  1EBPA                 77    0.353  50.077   0.448   0.022   0.552   0.426
E T  1EBPA                 78    0.326  45.230  -0.652   0.021   0.279   0.699
E A  1EBPA                 79    0.600  66.164  -1.006   0.019   0.141   0.840
E D  1EBPA                 80    0.559  80.509  -1.098   0.020   0.205   0.775
B T  1EBPA                 81    0.250  34.633  -1.410   0.005   0.262   0.733
E S  1EBPA                 82    0.380  44.501  -0.958   0.005   0.336   0.660
E S  1EBPA                 83    0.413  48.380  -0.463   0.005   0.336   0.660
E F  1EBPA                 84    0.289  58.042  -1.168   0.004   0.420   0.576
B V  1EBPA                 85    0.221  34.029   0.099   0.003   0.718   0.279
E P  1EBPA                 86    0.365  51.836   0.546   0.001   0.900   0.099
B L  1EBPA                 87    0.047   8.642   0.943   0.001   0.959   0.040
B E  1EBPA                 88    0.150  26.257   0.878   0.001   0.959   0.040
B L  1EBPA                 89    0.015   2.765   1.580   0.001   0.959   0.040
B R  1EBPA                 90    0.129  29.655   1.075   0.001   0.959   0.040
B V  1EBPA                 91    0.016   2.536   1.326   0.001   0.959   0.040
E T  1EBPA                 92    0.251  34.855   1.187   0.001   0.959   0.040
B A  1EBPA                 93    0.087   9.620  -0.464   0.001   0.900   0.099
E A  1EBPA                 94    0.313  34.526  -0.322   0.004   0.514   0.481
E S  1EBPA                 95    0.443  51.920  -0.732   0.021   0.279   0.699
E G  1EBPA                 96    0.567  44.623  -1.879   0.018   0.088   0.893
E A  1EBPA                 97    0.499  54.946  -1.766   0.018   0.088   0.893
E P  1EBPA                 98    0.503  71.376  -1.608   0.004   0.197   0.799
E R  1EBPA                 99    0.518 118.599  -1.527   0.021   0.279   0.699
E Y  1EBPA                100    0.394  84.198  -1.183   0.020   0.205   0.775
E H  1EBPA                101    0.542  98.608  -1.428   0.020   0.205   0.775
E R  1EBPA                102    0.489 112.004  -0.608   0.022   0.359   0.619
E V  1EBPA                103    0.361  55.501  -0.673   0.004   0.514   0.481
B I  1EBPA                104    0.128  23.680   0.366   0.004   0.616   0.381
E H  1EBPA                105    0.456  83.019   0.906   0.022   0.552   0.426
B I  1EBPA                106    0.097  18.037  -0.373   0.021   0.451   0.528
E N  1EBPA                107    0.513  75.074  -0.211   0.021   0.451   0.528
E E  1EBPA                108    0.466  81.393  -0.564   0.021   0.451   0.528
E V  1EBPA                109    0.294  45.203   0.262   0.022   0.552   0.426
B V  1EBPA                110    0.166  25.591  -0.612   0.022   0.552   0.426
E L  1EBPA                111    0.393  71.940   0.802   0.004   0.420   0.576
B L  1EBPA                112    0.220  40.337  -0.793   0.004   0.138   0.858
E D  1EBPA                113    0.543  78.232  -0.636   0.005   0.015   0.979
B A  1EBPA                114    0.251  27.682  -0.070   0.005   0.015   0.979
B P  1EBPA                115    0.142  20.107  -1.428   0.018   0.019   0.964
E V  1EBPA                116    0.441  67.812  -0.527   0.004   0.197   0.799
E G  1EBPA                117    0.509  40.074  -0.242   0.004   0.514   0.481
B L  1EBPA                118    0.117  21.514   1.529   0.003   0.718   0.279
E V  1EBPA                119    0.397  61.050   0.631   0.001   0.900   0.099
B A  1EBPA                120    0.111  12.254   0.057   0.001   0.900   0.099
E R  1EBPA                121    0.437 100.004   0.986   0.001   0.959   0.040
E L  1EBPA                122    0.372  68.113  -0.342   0.001   0.900   0.099
E A  1EBPA                123    0.324  35.683  -0.368   0.003   0.718   0.279
E D  1EBPA                124    0.589  84.860  -1.868   0.005   0.262   0.733
E E  1EBPA                125    0.585 102.130  -0.628   0.004   0.085   0.910
E S  1EBPA                126    0.468  54.908  -0.357   0.005   0.045   0.951
E G  1EBPA                127    0.386  30.394  -0.485   0.004   0.138   0.858
B H  1EBPA                128    0.242  43.965   0.976   0.003   0.718   0.279
B V  1EBPA                129    0.031   4.734   0.212   0.001   0.900   0.099
B V  1EBPA                130    0.209  32.200   1.379   0.006   0.962   0.032
B L  1EBPA                131    0.023   4.230   1.049   0.006   0.962   0.032
B R  1EBPA                132    0.164  37.579   0.676   0.011   0.918   0.071
B W  1EBPA                133    0.054  13.107  -0.160   0.001   0.900   0.099
E L  1EBPA                134    0.426  77.964   0.564   0.004   0.616   0.381
E P  1EBPA                135    0.324  46.004   0.149   0.004   0.138   0.858
B P  1EBPA                136    0.205  29.118  -0.484   0.005   0.045   0.951
E P  1EBPA                137    0.624  88.617  -1.154   0.005   0.015   0.979
E E  1EBPA                138    0.466  81.375  -1.556   0.018   0.019   0.964
E T  1EBPA                139    0.600  83.165  -1.714   0.018   0.019   0.964
E P  1EBPA                140    0.504  71.518  -2.460   0.018   0.019   0.964
E M  1EBPA                141    0.408  81.701  -2.200   0.005   0.045   0.951
E T  1EBPA                142    0.599  83.026  -1.034   0.004   0.138   0.858
B S  1EBPA                143    0.260  30.484  -0.366   0.004   0.420   0.576
E H  1EBPA                144    0.271  49.240   0.199   0.002   0.816   0.182
B I  1EBPA                145    0.058  10.767  -0.006   0.001   0.959   0.040
E R  1EBPA                146    0.258  59.013   1.467   0.001   0.959   0.040
B Y  1EBPA                147    0.044   9.317   0.467   0.001   0.959   0.040
B E  1EBPA                148    0.169  29.472   1.204   0.001   0.959   0.040
B V  1EBPA                149    0.026   3.950   0.927   0.001   0.959   0.040
B D  1EBPA                150    0.233  33.633   1.378   0.001   0.959   0.040
B V  1EBPA                151    0.102  15.724   0.788   0.001   0.959   0.040
E S  1EBPA                152    0.257  30.085   1.383   0.001   0.959   0.040
E A  1EBPA                153    0.472  52.036   0.952   0.002   0.816   0.182
E G  1EBPA                154    0.582  45.843   0.468   0.005   0.262   0.733
E N  1EBPA                155    0.702 102.802  -0.278   0.005   0.045   0.951
E G  1EBPA                156    0.684  53.870  -0.548   0.005   0.015   0.979
E A  1EBPA                157    0.612  67.442   0.138   0.018   0.019   0.964
E G  1EBPA                158    0.538  42.372   0.313   0.018   0.088   0.893
E S  1EBPA                159    0.525  61.530   0.683   0.022   0.359   0.619
E V  1EBPA                160    0.366  56.285   0.391   0.003   0.718   0.279
E Q  1EBPA                161    0.370  66.046   0.558   0.002   0.816   0.182
E R  1EBPA                162    0.515 118.027   0.607   0.001   0.900   0.099
E V  1EBPA                163    0.400  61.495   0.425   0.001   0.959   0.040
E E  1EBPA                164    0.398  69.531   0.146   0.001   0.959   0.040
E I  1EBPA                165    0.415  76.738  -0.840   0.002   0.816   0.182
E L  1EBPA                166    0.449  82.285  -0.662   0.004   0.514   0.481
E E  1EBPA                167    0.543  94.827  -0.383   0.004   0.197   0.799
E G  1EBPA                168    0.654  51.486  -0.601   0.005   0.045   0.951
E R  1EBPA                169    0.551 126.225  -0.180   0.005   0.262   0.733
B T  1EBPA                170    0.234  32.456   0.197   0.003   0.718   0.279
E E  1EBPA                171    0.391  68.238   0.661   0.001   0.900   0.099
B C  1EBPA                172    0.080  11.260   0.533   0.001   0.959   0.040
E V  1EBPA                173    0.341  52.381   1.421   0.001   0.959   0.040
B L  1EBPA                174    0.038   6.885   0.111   0.002   0.816   0.182
E S  1EBPA                175    0.504  59.022   0.605   0.004   0.420   0.576
E N  1EBPA                176    0.512  74.986  -0.389   0.004   0.138   0.858
B L  1EBPA                177    0.092  16.790  -0.002   0.005   0.045   0.951
E R  1EBPA                178    0.481 110.172   1.136   0.005   0.045   0.951
E G  1EBPA                179    0.473  37.225  -0.222   0.005   0.015   0.979
E R  1EBPA                180    0.627 143.583  -1.331   0.005   0.015   0.979
B T  1EBPA                181    0.211  29.266   0.338   0.004   0.197   0.799
E R  1EBPA                182    0.436  99.798   1.548   0.002   0.816   0.182
B Y  1EBPA                183    0.074  15.814   0.890   0.001   0.959   0.040
B T  1EBPA                184    0.226  31.291   2.062   0.001   0.959   0.040
B F  1EBPA                185    0.018   3.693   1.164   0.001   0.959   0.040
B A  1EBPA                186    0.100  11.075   1.237   0.001   0.959   0.040
B V  1EBPA                187    0.015   2.305   1.477   0.001   0.959   0.040
B R  1EBPA                188    0.083  18.961   0.898   0.001   0.959   0.040
B A  1EBPA                189    0.015   1.620   1.039   0.001   0.959   0.040
B R  1EBPA                190    0.151  34.510   1.092   0.002   0.816   0.182
B M  1EBPA                191    0.179  35.858  -0.850   0.004   0.616   0.381
E A  1EBPA                192    0.358  39.496  -0.323   0.005   0.262   0.733
E E  1EBPA                193    0.584 101.972  -2.114   0.004   0.085   0.910
E P  1EBPA                194    0.474  67.289  -1.954   0.018   0.088   0.893
E S  1EBPA                195    0.482  56.467  -2.023   0.005   0.045   0.951
E F  1EBPA                196    0.355  71.188  -1.626   0.005   0.045   0.951
E G  1EBPA                197    0.557  43.820  -0.881   0.005   0.045   0.951
B G  1EBPA                198    0.269  21.170  -1.664   0.005   0.045   0.951
E F  1EBPA                199    0.312  62.719  -0.350   0.019   0.141   0.840
B W  1EBPA                200    0.114  27.513  -0.684   0.018   0.088   0.893
B S  1EBPA                201    0.086  10.114  -1.564   0.018   0.047   0.935
E A  1EBPA                202    0.502  55.276  -0.226   0.018   0.047   0.935
B W  1EBPA                203    0.191  45.839  -0.850   0.018   0.047   0.935
B S  1EBPA                204    0.190  22.303  -0.901   0.018   0.047   0.935
E E  1EBPA                205    0.493  86.110  -0.048   0.004   0.197   0.799
E P  1EBPA                206    0.469  66.509  -0.357   0.004   0.514   0.481
B V  1EBPA                207    0.237  36.350   0.906   0.002   0.816   0.182
E S  1EBPA                208    0.463  54.240   0.594   0.001   0.900   0.099
B L  1EBPA                209    0.185  33.947  -0.480   0.001   0.900   0.099
E L  1EBPA                210    0.432  79.081   0.857   0.001   0.900   0.099
E T  1EBPA                211    0.546  75.800  -1.461   0.005   0.015   0.979

Explain the output.  Go back.
```

---
